# The intrapelvic approach to the acetabulum

**DOI:** 10.1007/s00402-024-05667-x

**Published:** 2024-12-18

**Authors:** Axel Gänsslen, Mario Staresinic, Dietmar Krappinger, Jan Lindahl

**Affiliations:** 1 Department of Trauma Surgery, Hannover Medical School, Hannover, Germany; 2 Department of Trauma and Orthopedics, Johannes Wesling Hospital, Minden, Germany; 3 Clinic for Surgery, Department of General and Sports Traumatology, University Hospital “Merkur” Zagreb, Zagreb, Croatia; 4 Department of Orthopaedics and Traumatology, Medical University of Innsbruck, Innsbruck, Austria; 5 Department of Orthopaedics and Traumatology, Helsinki University Hospital, University of Helsinki, Helsinki, Finland

**Keywords:** Intrapelvic approach, Stoppa´s hernia repair, Results, Extended intrapelvic approach

## Abstract

The today well accepted intrapelvic approach for acetabular and pelvic ring injury fixation was first described by Hirvensalo and Lindahl in 1993 followed by a more detailed description by Cole and Bolhofner in 1994. Compared to the well-known ilioinguinal approach, described by Letournel, this approach allows an intrapelvic view to the medial acetabulum, while using the ilioinguinal approach a more superior, extrapelvic view, is dissected to the area of the acetabulum. Several names have been used to describe the new intrapelvic approach with increasing usage, mainly ilio-anterior approach, extended Pfannenstiel approach, Stoppa-approach, Rives-Stoppa approach, modified Stoppa approach and recently anterior intrapelvic approach. Especially names including “Stoppa”, based on the French surgeon Rene Stoppa, an inguinal hernia surgeon, have been discussed. In contrast to the presently used intrapelvic approach, the original the Rives-Stoppa approach refers to a sublay-retromuscular technique, which places a mesh posterior to the rectus muscle and anterior to the posterior rectus sheath without dissecting along the upper pubic ramus. Thus, intrapelvic approach is not a Rives-Stoppa approach. The Cheatle-Henry approach, another inguinal hernia approach, refers best to the presently used intrapelvic approach. Discussing the anatomy and the different dissections, this approach allows anteromedial access to the anterior column and a direct view from inside the true pelvis to the quadrilateral plate and medial side of the posterior column. Thus, we favor to use the term “Intrapelvic Approach”.

## Introduction

Based on the tremendous work by Emile Letournel and Robert Judet, several limited and extended approaches addressing open reduction and internal fixation of acetabular fractures became standard in the 60ies, 70ies, and 80ies [29, 40, 41] e.g.:

- Kocher-Langenbeck approach.
- ilioinguinal approach.
- iliofemoral approach.
- extended iliofemoral approach.

Treatment of acetabular fractures became of increasing relevance since the 1960ies, when Emile Letournel developed a new approach especially to anterior fractures of the acetabulum – the ilioinguinal approach [29, 38, 39].

Especially, the second window offers a different access to the anterior border of the bone from the iliopectineal eminence as far as to the middle of the superior pubic ramus, the middle part of the pelvic brim and digital access to the quadrilateral surface [29, 40], compared to the Smith-Peterson approach [65] and the iliofemoral approach [42].

The primary view of the ilioinguinal is from above the iliopectineal line, e.g. the pelvic brim. Thus, the ilioinguinal can be considered as an extrapelvic approach (at the level of the joint).

### The Helsinki approach

The Helsinki intrapelvic approach was developed in the late 80ies for stabilization of displaced pubic rami and some acetabular fractures [20]. At this time, after a decade of external fixation, open reduction and fixation of pelvic ring injuries focused primarily on symphyseal and SI-joint reconstruction.

With the so-called “ilio-anterior approach” even displaced bony fractures could be addressed [22].

With this new anterior approach, dissection was performed using either a longitudinal skin incision or a classical Pfannenstiel incision. Dissection along the pelvic brim was performed up to the SI-joint and the complete inner surface of the true hemipelvis could be visualized. If necessary, the 1st window of the ilioinguinal approach was opened for dissection to the SI-joint and lateral sacrum [22, 23].

As a main advantage, subperiosteal dissection without relevant muscle detachment was proposed, resulting in less blood loss, shorter OR-times and rarely complications.

One year later in 1994, Cole and Bolhofner again described this approach, focusing on acetabular fracture stabilization [12]. They first called this approach “extended Pfannenstiel approach” but the name was changed to “Stoppa-approach“, when discussing older literature from hernia repair.

The Helsinki approach was not addressed in their paper, but the (more detailed) description of the approach was comparable to the “ilio-anterior intrapelvic approach” of the Helsinki group.

In 1993, a new approach for pelvic ring and acetabular fractures was introduced by the Helsinki group. Several names were used with increasing experience: (1) ilio-anterior approach, (2) extended Pfannenstiel approach, (3) Stoppa-approach.

### Is the Helsinki intrapelvic approach a Rives-Stoppa approach?

The original Stoppa approach is ascribed back to the work by Rene Stoppa and Jean Rives. The classic Rives-Stoppa repair of ventral incisional hernias is based on a retromuscular (rectus abdominus muscle) placement of a mesh anterior to the posterior rectus abdominus fascia and primary closure of the anterior fascia [67–71].

The extraperitoneal approach addressing femoral hernias is returned to Lenthal Cheatle [8]. He wrote in 1920:

The patient is placed in the Trendelenburg position, an incision is made to one side of the middle line, the rectus abdominis is split longitudinally, and the abdominal wall is retracted to the side of the operation. The peritoneum is smoothed away by a dry swab from the parietes. The operation is conducted without opening the peritoneum.

In a further description in 1921 [9], Cheatle changed the approach and described the approach as followed:

The patient is placed in the Trendelenburg position, and the operator stands on the side opposite the hernia. A transverse skin incision 4 or 5 inches long is made 1 1/2 inches above the symphysis pubis. Its centre corresponds with the middle line. A transverse incision is made in the aponeurosis of the rectus abdominis of both sides, care being taken not to injure either linea semilunaris. The linea alba is undercut upwards and downwards, to within one or two inches of the umbilicus, and to the symphysis respectively: in doing so the sheath of each pyramidalis muscle will be opened. The opening thus made in the aponeurosis is retracted up and down and the subperitoneal tissue exposed by separating the abdominal muscles in the middle line. The peritoneum and its contents are pushed up on both sides, and if necessary kept up by packing.

**Fig. 1:**
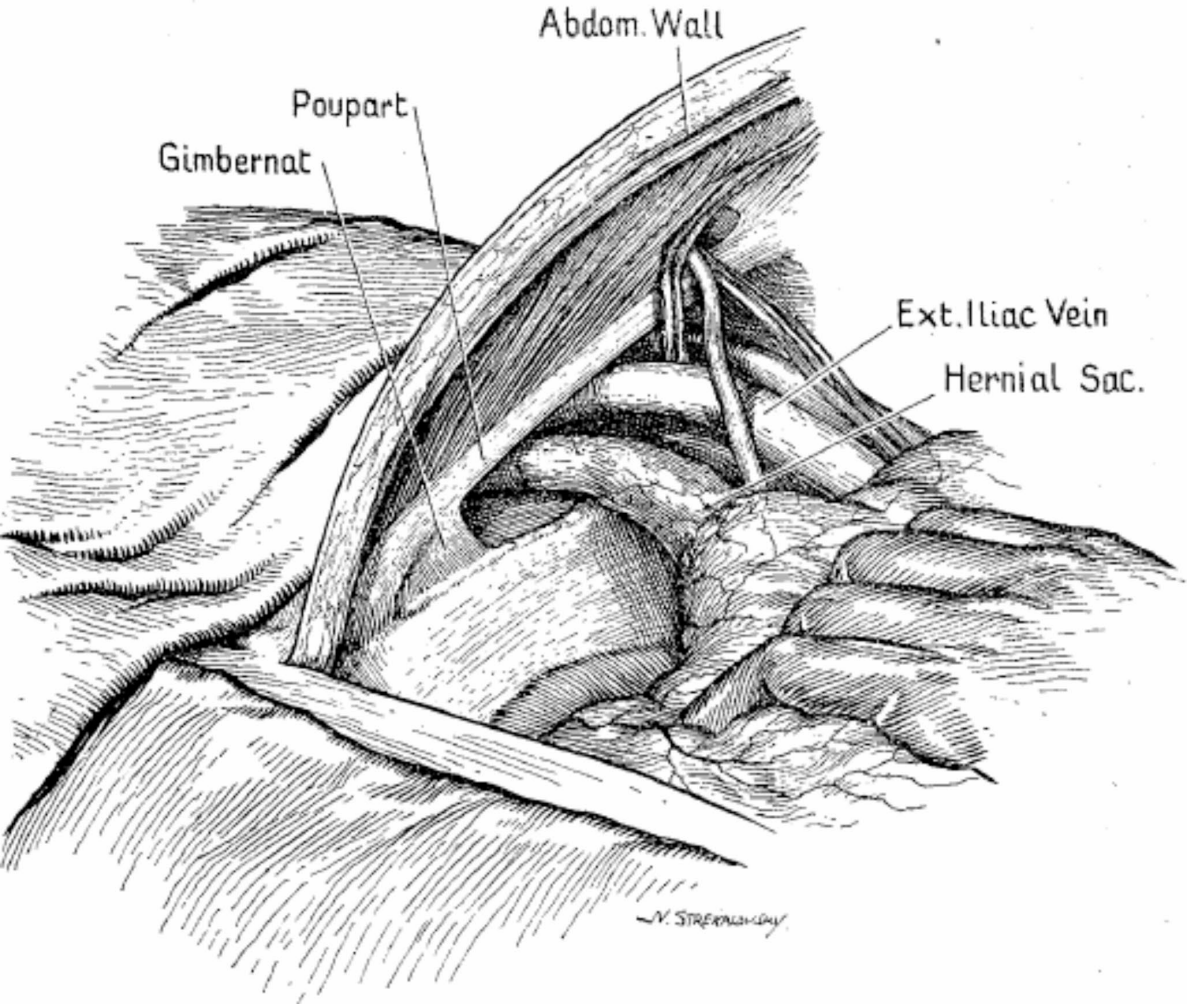
Drawing from Henry showing the access to the superior ramus [19]

Henry in 1936 described already an extraperitoneal approach using a midline incision [19]:

through a midline incision, I separated the recti at and below the navel, and stripped the unopened peritoneum from the sides of the bladder and from the pelvic wall… After retracting the external iliac vein outwards (Fig. 1).

The Cheatle-Henry approach refers best to the intrapelvic approach described by Hirvensalo, Lindahl and Böstman.

**Fig. 2:**
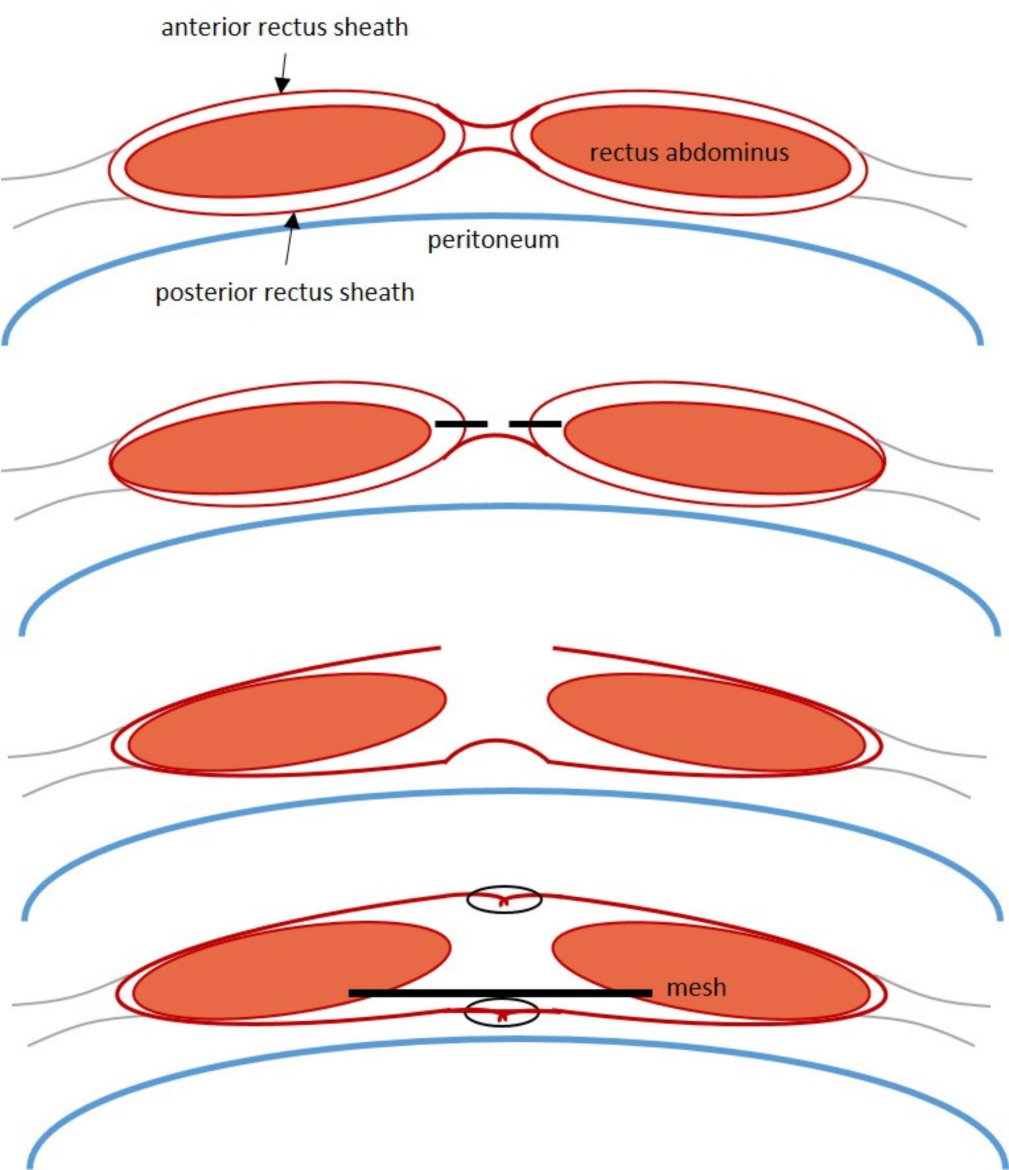
Schematic concept of the Rives-Stoppa retromuscular approach

It is the merit of René Stoppa and Jean Rives to repair the transversalis fascia in the preperitoneal space of the groin by a synthetic mesh [69, 71]. In a detailed description, Stoppa et al. reported [70]:

“The patient is placed on his back on a standard operating table, in a light Trendelenburg position; the surgeon is on the opposite side of the hernia. An adhesive field is applied as the usual protection against contamination of skin origin. A median subumbilical incision is made, subcutaneous tissue and fascia umbilico-prevesicalis are cut with Mayo scissors, and at the superior portion of the incision the Richet’s fascia umbilicalis is maintained. The retroparietal dissection is then done; we advise starting from the lower portion on the median line in the Retzius retropubic space; then proceed laterally, dissecting the posterior portion of the rectus abdominis muscle on the far side of the operator, using, in succession, a small Farabeuf retractor and a short straight valve, proceeding behind the epigastric vessels in the Hesselbach ligament. The cleavage then proceeds downward in front of the bladder up to the prostatic compartment, then outward and posteriorly behind the superior iliopubic ramus in Bogros’s space. Thus, the hernial pedicle on the side opposite the operator is isolated (Fig. 2), as is the spermatic cord that is either united or freed from the hernial sac, depending on the nature of the hernia. The iliopsoas muscle and external iliac vessels and their tunics are readily and safely exposed. Dissection of the hernial pedicle does not necessitate any difficult search for the different elements, even in multi-recurring hernias.”.

The basic principles of the Rives-Stoppa retromuscular approach include [7, 50]:

- midline incision of the skin.
- dissection of the subcutaneous fat.
- identification of the linea alba.
- median incision of the linea alba.
- vertical incision of the rectus sheath along the entire length of the incision.
- dissection of the posterior rectus sheath: retromuscular plane between the muscle belly and the posterior sheath.

Dissection typically ends at the upper level of the pubic rami. No dissection is performed along the linea terminalis to visualize the corona mortis or the iliac external vessels or the obturator neurovascular bundle [53, 55] and no fixation of the mesh is routinely performed [67].

Overall, the Rives-Stoppa approach is originally a sublay-retromuscular technique, which places a mesh posterior to the rectus muscle and anterior to the posterior rectus sheath [3, 52]. No dissection is performed along the pubic ramus or in the true pelvis [6].

### Combined and modified approaches

Karunakar et al. described the combination of the ilioinguinal and intrapelvic approach with the advantage of better visualization of the medial wall “below” the terminal line [30]. Mobilizing the complete ipsilateral rectus belly, plates can be placed from the inner side of the pelvis against central fracture displacement. This modification overlaps to the dissection of the pure intrapelvic approach.

Karunakar et al. in 2004 combined the classical ilioinguinal approach with the intrapelvic approach to visualize the quadrilateral surface from extra- and intrapelvic [30].

In 2019, Mayo and Unno described a modified ilioinguinal approach [47]. They incorporated “a modified medial window that limits dissection around the external iliac vessels, expands surgical exposure of the anterior pelvic ring, and provides additional reduction possibilities while preserving the capabilities of the lateral and middle windows” [47]. Beside the classical ilioinguinal approach, they integrated the technique of the classical intrapelvic approach [21, 23] through a vertical midline incision or extension of the skin incision to the opposite side of the pubic symphysis. In an overview article in 1990, this modification was not yet recommended [46].

### Recent discussions on the intrapelvic approach

With ongoing experience using the intrapelvic approach, it was called Rives-Stoppa Approach, modified Stoppa Approach or Anterior Intrapelvic Approach or only Intrapelvic Approach [15, 16, 21, 57].

As this approach allows a direct view from inside the true pelvis, the term “Intrapelvic Approach” should be used. As no posterior intrapelvic approach exists, the term “anterior” should be avoided.

The detailed surgical technique is now standardized [15, 16, 21, 72].

Compared to the original ilioinguinal approach, systematic reviews in 2017 identified several advantages:

- higher rates of anatomical reductions [48].
- shorter operative time [48, 77].
- no significant differences in terms of functional outcomes.
- no significant differences in blood loss.

While Meena et al., reported a lower complication rate and no differences in intraoperative blood loss, Wang et al. found no differences regarding early and late complications, but a reduced blood loss in their analysis [48, 77]. The overall functional results were comparable with both approaches [48, 77].

Five years later, recent meta-analyses and systematic reviews from 2022 showed comparable results [63, 66]:

- shorter duration of surgery.
- lesser number of overall complications.
- less intraoperative blood loss.
- lower rates of infection.
- less intraoperative vascular injuries.
- less intraoperative nerve injuries.
- less development of heterotopic ossification.
- comparable functional outcome.

The quality of reduction rate was controversial between these studies [63, 66].

### Approach-specific results

The following analysis gives an overview on approach-specific data, e.g. blood loss, time of surgery and approach-specific complications. A fracture-type specific analysis is not possible, as the majority of papers summarize different fracture-types.

Overall, data of 1882 patients are reported in the literature since 2007 dealing with the intrapelvic approach.

Only few studies deal with an isolated intrapelvic approach [1, 13, 28, 35, 36, 44, 60, 74] or with the combined intrapelvic approach + 1st window incision [2, 14, 17, 26, 32, 33, 37, 43, 51, 56, 59, 73], while the majority of reports included cases with an additional Kocher-Langenbeck approach [4, 11, 18, 24, 27, 31, 34, 64, 75, 76, 82, 84, 85] or the Smith-Petersen approach [5, 57, 80, 81] or both [61] into the overall analysis of intrapelvic approaches. In some reports, it was not specified or no data were available, how many additional incisions/approaches were performed [10, 23, 25, 45, 49, 54, 58, 62, 78, 79, 83, 85].

#### Quality of reduction

No data are available on quality of reduction for specific fracture-types. Thus, the majority of reports analyze a mixture of typical fracture types, where an IP approach is suitable.

Many of the published papers report on quality of reduction [2, 4, 5, 14, 17, 18, 23–25, 27, 28, 32, 33, 35–37, 43, 45, 49, 51, 57, 58, 60, 61, 64, 73–76, 80–82, 84].

The exploratory analysis of these data shows a rate of anatomic reductions in 66.1%.

#### Blood loss and surgery time

Data on blood loss [1, 13, 35, 44, 74] and surgery time [1, 35, 44, 60, 74] after isolated IP-approaches are available from overall six studies.

The average blood loss could be calculated from 159 patients to be 912.6 ml (range: 277.1-1850 ml), while the average surgery time in 184 patients was 175.8 min (range: 146–221 min).

Additional data are available for patients with an additional opening of the 1st window of the ilioinguinal approach (= extended IP approach) [2, 14, 17, 26, 32, 33, 37, 43, 51, 59, 73]. Three studies did not distinguish in how many cases the additional incision was used [25, 49, 58]. Only in two studies, all patients had an extended AP approach [37, 59], while in the other nine studies, the mean rate of additional extended IP approaches was 61.7% [2, 14, 17, 26, 32, 33, 43, 51, 73].

The average blood loss in 42 patients with a full extended IP approach was 856.5 ml, while the average surgery time in 184 patients was 203.5 min.

The average blood loss in the group of patients with the IP approach and 61.7% additional 1st window incision was 700.5 ml, while the average surgery time was 185.4 min.

#### Neurovascular complications

In 36 reports, information is available regrading neurovascular complications [2, 4, 10, 11, 13, 14, 17, 18, 23–27, 31–33, 35–37, 43–45, 49, 56–58, 60–62, 64, 73–75, 80–82, 84].

Overall, 1482 approaches (different combinations: isolated IP, extended IP, IP + Kocher-Langenbeck, IP + Smith-Peterson) can be analyzed. The following complication rates could be calculated:

- 2.16% vascular damage.
- 0.07% femoral nerve lesion.
- 2.97% obturator nerve lesion.
- 2.56% lateral cutaneus femoral nerve lesions (only in cases with an additional 1st window approach).
- 0.34% sciatic nerve lesions (2/5 in isolated IP approaches during manipulation of the posterior column fracture; 3/5 with additional Kocher-Langenbeck approach).

Of 30 vascular lesions, the predominant type of vascular lesion was iliac external vein and obturator artery injury (3x corona mortis bleeding, 2x superior gluteal artery (1x additional Kocher-Langenbeck approach), 9x external iliac vein, 2x femoral vein, 4x obturator artery, 1x arterial thrombosis of external iliac artery, 9x not specified).

#### Soft-tissue complications

In 37 reports, information is available regarding soft-tissue complications [2, 4, 10, 11, 13, 14, 17, 18, 23–27, 31–34, 36, 37, 43–45, 49, 56–58, 60, 61, 64, 73–75, 80–82, 84].

Hematoseroma were observed in 0.8%, A superficial wound infection was reported in 1.89% and deep infections in 2.35%. The latter were treated by surgical debridement in the majority of cases.

#### Thromboembolic complications

Using the IP approach, there is a potential risk of developing deep vein thrombosis and/or pulmonary embolism (DVT/PE) due to manipulation of the external iliac, obturator and rarely the internal iliac vessels.

The reported DVT rate analyzing 1882 reported IP approaches was 1.91% [2, 11, 14, 23, 26, 32, 33, 44, 45, 56, 57, 75, 80, 82] and the corresponding rate of PE was 0.27% [13, 23, 32].

#### Heterotopic ossifications

The rate of heterotopic ossifications after an IP approach was 1.38% [2, 5, 23, 24, 34, 37, 61, 81]. All ossifications were of minor grade according to Brooker.

#### Hernias

Seven hernias (0.37%) are reported after analysis of 1882 patients with an IP approach [2, 14, 27, 32, 57].

### Data on isolated IP approaches

Only seven reports dealed with isolated IP approaches [1, 13, 28, 35, 44, 60, 74].

Not in every report, data of all parameters were available. Thus, the following analysis only gives exploratory results:

- 912.65 ml mean blood loss.
- 175.80 min mean surgery time.
- 45.63% anatomic reductions.
- 1.9% vascular injuries.
- 3.37% obturator nerve lesions.
- 1.12% sciatic nerve lesions.
- 4.13% hematoseroma.
- 9.26% superficial infections.
- 1.09% deep infections.

No injuries to the femoral nerve, and the lateral cutaneus femoral nerve were reported. No thromboembolic complications, no heterotopic ossifications and no hernias occured.

### Data on combined IP and the 1st window approaches

Several reports stated, that an additional 1st window approach was performed, but not in every case [2, 14, 17, 26, 32, 33, 37, 43, 51, 56, 59, 73]. The overall rate of an additional 1st window approaches was 56.5%.

Analyzing only reports with more than 75% additional 1st window approaches left five studies with overall 92.6% true extended IP approaches [14, 32, 37, 43, 59]. Again, not in every report, data of all parameters were available. The following analysis gives an adequate overview of the extended IP approach-specific results:

- 1008.92 ml mean blood los.
- 144.59 min mean surgery time.
- 65.81% anatomic reductions.
- 1.29% vascular injuries.
- 1.23% obturator nerve lesions.
- 1.82% lateral cutaneus femoral nerve lesions.
- 3.87% deep infections.
- 2.58% DVT.
- 2.73% PE.
- 3,7% heterotopic ossifications.
- 2.38% hernias.

No injuries to the femoral and sciatic nerve were reported. No superficial infections and hemato-seroma were observed.

## Conclusion

The “Intrapelvic Approach”, first described by the Helsinki group, is a true intrapelvic approach, which allows dissection within the true pelvis. It is a technically easy approach and is today most commonly used to treat acetabular fractures with a predominant anterior fracture pathology. Even simple concomitant posterior column fractures and transverse fracture components can be easily addressed. The approach-specific perioperative results are more favorable, than using the classical ilioinguinal approach.

## Declarations

### Level of evidence

V
